# Supplementary material for: Evolution of strain diversity and virulence factor repertoire in pediatric Staphylococcus aureus isolates
Source: PLoS One. 2025 Jul 31;20(7):e0326353. doi: 10.1371/journal.pone.0326353 (PMC12312961; doi:10.1371/journal.pone.0326353)
Supplement: Attachment — Submitted filename: May24_Response to Reviewers1 (1).docx [file pone.0326353.s005.docx]

*Make sure to address all asterisks before submitting*.

Reviewer's Responses to Questions

**Comments to the Author**

1. Is the manuscript technically sound, and do the data support the conclusions?

The manuscript must describe a technically sound piece of scientific research with data that supports the conclusions. Experiments must have been conducted rigorously, with appropriate controls, replication, and sample sizes. The conclusions must be drawn appropriately based on the data presented.

Reviewer #1: Yes

Reviewer #2: Yes

Reviewer #3: Partly

2. Has the statistical analysis been performed appropriately and rigorously?

Reviewer #1: Yes

Reviewer #2: Yes

Reviewer #3: Yes

3. Have the authors made all data underlying the findings in their manuscript fully available?

The [PLOS Data policy](http://www.plosone.org/static/policies.action#sharing) requires authors to make all data underlying the findings described in their manuscript fully available without restriction, with rare exception (please refer to the Data Availability Statement in the manuscript PDF file). The data should be provided as part of the manuscript or its supporting information, or deposited to a public repository. For example, in addition to summary statistics, the data points behind means, medians and variance measures should be available. If there are restrictions on publicly sharing data—e.g. participant privacy or use of data from a third party—those must be specified.

Reviewer #1: Yes

Reviewer #2: Yes

Reviewer #3: Yes

4. Is the manuscript presented in an intelligible fashion and written in standard English?

PLOS ONE does not copyedit accepted manuscripts, so the language in submitted articles must be clear, correct, and unambiguous. Any typographical or grammatical errors should be corrected at revision, so please note any specific errors here.

Reviewer #1: Yes

Reviewer #2: Yes

Reviewer #3: Yes

5. Review Comments to the Author

Please use the space provided to explain your answers to the questions above. You may also include additional comments for the author, including concerns about dual publication, research ethics, or publication ethics. (Please upload your review as an attachment if it exceeds 20,000 characters)

**Reviewer #1:** This is an interesting assessment of pediatric S aureus isolates (MSSA and MRSA) at a single academic medical center. The authors reveal some changes in susceptibility (loss of macrolide efflux with increased susceptibility over time with clinda staying constant) and shift toward the 'big 2' CC8 and CC8 being responsible for more invasive infections.

While a genotypic analysis is helpful, expression of some key virulence exotoxins (eg alpha toxin) would be of interest. For example, while the strains all contain the alpha toxin gene, I suspect toxin expression will be variable over time. One might anticipate community-based infection to harbor more alpha-toxin producing strains while nosocomial strains might not express. For example, there is less need for exotoxin-driven invasion in the hospital when health care delivery has already granted easy access via wounds and catheters. The parsimony of evolution would be expected to relinquish the metabolic demand for such toxins in these settings.

The expression of alpha toxin can actually be done grossly through a crude hemolysis assay using beta-toxin disks

We agree that evaluation of alpha toxin expression would be an interesting addition to this manuscript. Unfortunately the bacterial isolates and case records for the study were accessible during a specific window of time which is now closed, and thus cannot be added to this dataset. Determining the presence of alpha toxin would be an excellent addition to future manuscripts. We appreciate this excellent point.

Minor comment-- Why were the catheter and foreign body infection isolates not sent for sequencing?

Thank you for the comment. Our aim with this analysis was to collect staphylococcal isolates that were able to invade without the aid of hardware or other perturbuations of the host-pathogen interface. As such, we aimed to collect only the most virulent invasive isolates (those isolates that could surpass an intact innate immune system), and therefore excluded immunocompromised patients or those with indwelling catheters, hardware, etc.

**Reviewer #2:** I read with interest the contribution by Free et al. (PONE-D-24-08440). The authors describe the phylogenetic drift in invasive pediatric Staphylococcus aureus isolates over the past decade. The manuscript was a pleasure to read with very clear writing. Its findings are well founded and introduced with the appropriate context of existing staphylococcal phylogeny knowledge. While I may question the use of neonatal isolates as a control to represent commensal isolates across the pediatric age spectrum, the authors clearly define this as a study limitation. All of my comments are minor and reflective of the superior skill of the authors to communicate their scientific findings. Of these, the most significant in my opinion would be to clarify the single-center/multi-center nature of the sample collection and, if multi-center, how many isolates were recovered from each site. Thank you for the opportunity to review this strong submission for publication with PLOSone. I would be happy to review any future revisions.

Identified Areas for Improvement:
*** In your conclusions section you state that sak and lukED were associated with invasive infection over time. What exactly do you mean by this? Based on your figures, sak and lukED were clearly associated with invasion (consistent with observations in the adult population) but I don’t see a clear temporal relationship (if anything, the prevalence of sak and lukED positivity appears to go down with time).**

Thank you for prompting this clarification. In response to this comment, we have clarified in the final paragraph of the manuscript that the two factors were persistent over time, which is more accurate as we did not intend to imply they were increasing over time. Thank you for the comment.

* A recent study specifically identified sak as the genetic element most predictive of invasion and poor clinical outcome. In addition to including this reference, **it might add interest to note that sak is typically localized to a prophage, specifically one that also bears an immune evasion cluster, is suspected to be easy to mobilize and potentially able to persist as an episome. This would conceivably facilitate its dissemination between populations and conversion of commensal populations into invasive ones.**

Thank you for this comment. We have now added several lines of discussion around this excellent point, now present in the revised manuscript, Lines 331-336 of “Manuscript,” 383-388 of “Revised Manuscript with Track Changes”:

Sak is located on a prophage that contains an immune evasion cluster, and its presence on a prophage could allow for potential spread from invasive to commensal populations. Wang et al found an association between staphylokinase and invasive human infection when comparing rates of staphylokinase carriage in livestock-associated S. aureus to human-adapted staph aureus. They also observed milder pneumonia in mice infected with sak gene knockout isolates.

* Please modify the date range for column 2 in table 2 for accuracy.

Thank you for pointing this out; we regret this error. This has been addressed.

* Please comment on the breakdown into different date ranges. Why does the first cohort span six years, the second five and the third only four? Was there a reason for the differences?

We endeavored to look at changes over time and wanted roughly equal numbers in each cohort. The 2010-2012 group included 45 isolates, the 2014-2017 group included 40 isolates, and the 2018-2022 included 35 isolates. At our institution, we observed a decline in invasive *S. aureus* infections during the pandemic. We sought to make cohorts with similar amounts of isolates over similar periods of time.

* Please comment on the geographical distribution of your collection. Were all pediatric cases collected at CHOP? VUMC? If it is multi-center, that may enrich your phylogenetic distribution, although your controls seem to have been only collected at a single center.

Regardless, a more robust discussion of the study sites and their strengths/limitations is warranted. I would also consider a statement about the external validity of your study – the results are representative of isolates from the Eastern United States; I’m not certain they would represent pediatric isolates in other areas of the world.

Thanks for this comment, and we agree this is an important point. This was a single center study at Vanderbilt University Medical Center, and we have clarified this in two places in the manuscript: in the Methods section, indicating a single-center study, and in the Discussion section, noting that our observations in phylogenetic drift may not be generalizable to areas outside this geographic area, as the reviewer suggests.

* Regarding graphs and figures, please consider a color scheme that allows for differentiation when printed in black-and-white.

Unfortunately it was quite challenging to find any color scheme that would provide enough shades for all of the components of figures 1 and 2; we have attempted to find one that still allows differentiation when printed in black and white but acknowledge it is still difficult.

* I may have missed this, but if you haven’t submitted your sequence reads, contigs or assembled files to NCBI or EMBO, please consider doing so to more comprehensively satisfy the data availability requirements.

Isolate genomes have been submitted to PubMLST to evaluate sequence typing/clonal complex and are publically available. See attachment “Sequences submitted to PubMLST” for de-identified isolate names. A sentence has been added to the manuscript: “Consensus sequences with MLST assignments remain publicly available via PubMLST.”

**Reviewer #3:** This work describes the genetic evolution of S. aureus in pediatrics over the last 12 years (2010-2022). The methods are technically sound and well described, albeit the comparison of invasive infection vs colonization with different timeframes is weak. The findings from this study indicate a transition of S. aureus clonal type and pathogenicity over this time period. These data are new to the field as ongoing S. aureus genetic epidemiology is unique to the given situation. There are areas of the manuscript that require some attention including

1) The colonization timeline comparison (vs invasive infection) is difficult to justify and therefore make general comparisons. Some clarification/further details as to why the colonization cohorts were selected and different than then invasive group is needed.

NICU colonization isolates collected around 2012 were sent to another lab. Our lab no longer had access to those isolates. However we did have colonization isolates from 2004 and felt it would be helpful to include these as a comparison group. We agree that the comparison is imperfect and have attempted to note this in the manuscript.

2) There is good evidence that patients who are colonized with S. aureus and become infected have less severe acute infections. Were the patients from the invasive infection group tested for colonization as well? If yes, were they included or excluded? Further details on this as well as discussion of these known factors is warranted.

Thank you for this comment. Patients from the invasive infection group were not tested for colonization. This is because by the time the pediatric infectious diseases service was consulted or by the time the patient would had a positive blood culture (alerting our team to the possibility of an enrollment candidate), they were typically on anti-staphylococcal antibiotics for 48 hours or more. We opted not to test these patients for colonization as we anticipated a high rate of false negatives. A sentence addressing this important point has been added to the discussion section of the manuscript (lines 418-422 of “Manuscript with Track Changes, lines 365-366 of “Manuscript”).

3) The authors should provide a definition for severe immunocompromised as that is an exclusion factor in this study.

Thank you for this comment. The manuscript has been revised at the reviewer’s suggestion: “Patients with severe immunocompromised were excluded (example: a patient with relapsed acute myelogenous leukemia on chemotherapy with secondary neutropenia was excluded, a patient with cystic fibrosis and osteomyelitis was included, a patient with acute lymphoblastic leukemia in remission with a normal neutrophil count and pyomyositis was included).”

4) It is odd that mortality is not included in the disease severity. Although only 1 patient succumbed to mortality in the study, was this patient deemed severe?

Thank you for this clarification. Patient death is now included in disease severity score, and this patient has a severity score of 3.

5) For statistical analysis, the authors used Wilcoxon rank test as one of the tests. There is no indication that the data are nonparametric distribution or evaluated for data distribution for statistical testing.

Yes, we did not evaluate the distributional assumptions of our data before applying the Wilcoxon test.  This was an intentional choice because the Wilcoxon test does not assume Normality, or any other particular distributional assumption, for the data.  The Wilcoxon tests is based on the rank of the data points, which only requires an ordinality assumption that can be evaluated without having to look at the data.  In practical terms, this means that the Wilcoxon test can be used even when the data are not Normally distributed or when there are outliers that could potentially skew the results of a t-test.  We prefer this robust approach to data analysis when possible.  We have added a sentence to the Statistical analysis "The Wilcoxon test is a nonparametric test that is robust to departures from Normality, so we did not evaluate the data distribution prior to testing."

The P-values reported in Table 2 test for any difference across time period rather than doing all possible pairwise comparisons between groups.  We use this approach to limit multiple testing.  Significant results should be interpreted as, for example, "MRSA rates differed over time period", with the percentage given for the reader to evaluate how they differed. Other multiple degree of freedom tests are presented in the same manner for other tables (Table 5, Table 6).  This has been clarified in the Statistical Methods with the additional sentence "For categorical variables with more than two levels, we report the P-value testing the null hypothesis that all proportions are equal versus the alternative hypothesis that at least one proportion differs".

6) The range of data is not presented in some of the demographic data and tables. See table 3 for example.

Thank you for this feedback. Ranges have been added to the tables as suggested.

7) The authors should indicate comparisons for the P values in table 2. There are 3 time periods presented and P values for each.

For categorical variables with more than two levels, we report the P-value testing the null hypothesis that all proportions are equal versus the alternative hypothesis that at least one proportion differs. In table 2, P values greater than 0.05 indicate that antibiotic resistance did not change over time.

8) Table 2 shows a column of 2019-2014. This appears to be an error and should be corrected.

We regret this error. This has been corrected.

9) There are some additional susceptibility profiles that would be valuable to add a) vancomycin MIC in MRSA and MSSA as this has trended down over in the last 10+years and b) penicillin MIC in MSSA as evidence is emerging that S. aureus is becoming more penicillin susceptible. Both antibiotics would be valuable to include.

We agree that these additions would have been interesting and beneficial inclusions. However, unfortunately the case records for the study were accessible during a specific window of time which is now closed, and thus cannot be added to this dataset.

10) In the discussion, Lines 319-330 are highly speculative. The low number of isolates limits the extrapolation of the data to their hypotheses. It seems that the authors may be trying to fit their data to known assumptions without listing limitations

We agree with your feedback and regret the potential over-reach of conclusions; we have now removed these speculative lines to ensure accuracy of the conclusions.

11) There is discussion of staphylokinase as a potential target. However, how do the authors propose targeting staphylokinase when the clinical relevance is incompletely defined (Line 339)? These statements seem to contradict.

We concur with your feedback and have changed this paragraph.

6. PLOS authors have the option to publish the peer review history of their article ([what does this mean?](https://journals.plos.org/plosone/s/editorial-and-peer-review-process#loc-peer-review-history)). If published, this will include your full peer review and any attached files.


If you choose “no”, your identity will remain anonymous but your review may still be made public.


**Do you want your identity to be public for this peer review?** For information about this choice, including consent withdrawal, please see our [Privacy Policy](https://www.plos.org/privacy-policy).

Reviewer #1: No

Reviewer #2: **Yes:** Andrew David Berti

Reviewer #3: No

[NOTE: If reviewer comments were submitted as an attachment file, they will be attached to this email and accessible via the submission site. Please log into your account, locate the manuscript record, and check for the action link "View Attachments". If this link does not appear, there are no attachment files.]

While revising your submission, please upload your figure files to the Preflight Analysis and Conversion Engine (PACE) digital diagnostic tool, <https://pacev2.apexcovantage.com/>. PACE helps ensure that figures meet PLOS requirements. To use PACE, you must first register as a user. Registration is free. Then, login and navigate to the UPLOAD tab, where you will find detailed instructions on how to use the tool. If you encounter any issues or have any questions when using PACE, please email PLOS at [figures@plos.org](mailto:figures@plos.org). Please note that Supporting Information files do not need this step.
